# Oxidative Stress and Its Regulation in Diabetic Retinopathy

**DOI:** 10.3390/antiox12081649

**Published:** 2023-08-21

**Authors:** Cameron D. Haydinger, Genevieve F. Oliver, Liam M. Ashander, Justine R. Smith

**Affiliations:** College of Medicine and Public Health, Flinders University, Adelaide, SA 5042, Australia; cameron.haydinger@flinders.edu.au (C.D.H.); goliver@flinders.edu.au (G.F.O.); liam.ashander@flinders.edu.au (L.M.A.)

**Keywords:** oxidative stress, diabetes, diabetic retinopathy, macular edema

## Abstract

Diabetic retinopathy is the retinal disease associated with hyperglycemia in patients who suffer from type 1 or type 2 diabetes. It includes maculopathy, involving the central retina and characterized by ischemia and/or edema, and peripheral retinopathy that progresses to a proliferative stage with neovascularization. Approximately 10% of the global population is estimated to suffer from diabetes, and around one in 5 of these individuals have diabetic retinopathy. One of the major effects of hyperglycemia is oxidative stress, the pathological state in which elevated production of reactive oxygen species damages tissues, cells, and macromolecules. The retina is relatively prone to oxidative stress due to its high metabolic activity. This review provides a summary of the role of oxidative stress in diabetic retinopathy, including a description of the retinal cell players and the molecular mechanisms. It discusses pathological processes, including the formation and effects of advanced glycation end-products, the impact of metabolic memory, and involvements of non-coding RNA. The opportunities for the therapeutic blockade of oxidative stress in diabetic retinopathy are also considered.

## 1. Introduction

Diabetic retinopathy is considered to be caused principally by hyperglycemia. Its occurrence in humans is greatly diminished via intensive control of blood glucose [1,2]. Long-term systemic galactosemia recapitulates retinopathy in animal models, showing the direct involvement of hexose in the pathology [3,4]. One of the major cellular effects of hyperglycemia is elevated oxidative stress, which leads to upregulation of a range of pathological pathways [5]. This has led to significant research into the role of oxidative stress in the pathogenesis of diabetic retinopathy, among other diabetic complications, and therapeutic strategies to target processes that cause elevated oxidative stress. Despite much progress, unanswered questions remain, including the precise cellular and mechanistic sources of reactive oxygen species (ROS) in the diabetic retina, and a comprehensive understanding of the mechanisms that lead from oxidative stress to aspects of pathology.

Reactive oxygen species is a term referring to a range of molecules and radicals based around a reactive oxygen atom, including superoxide, hydrogen peroxide, and the hydroxyl radical, among others, each with unique sources, reactivity, and effects [6]. Certain experimental techniques and studies do not differentiate between the ROS that are measured, so the general term ROS is used in this review, as well as specific species when this is relevant or known. Oxidative stress describes the pathological state wherein elevated production of ROS causes damage to macromolecules, cells, and tissues.

Superoxide is the reactive species most implicated in initiating oxidative stress in diabetes. Other ROS and reaction products are also involved in pathological mechanisms generally downstream of superoxide production [7]. Retinas from diabetic mice produce more superoxide than retinas from control mice [8,9]. The same is true in rats [10]. Several stressors induce elevated production of superoxide, including hyperglycemia, hypoxia, and cytokines. Multiple retinal cell types are involved as sources of oxidative stress or as downstream targets of its effects in diabetes.

Oxidative stress is also involved in the pathophysiology of retinal diseases other than diabetic retinopathy, including age-related macular degeneration, retinitis pigmentosa, and retinopathy of prematurity [11,12,13]. These diseases differ in the causes of oxidative stress and the cell types most involved. For example, age, genetic factors, and oxygen dyshomeostasis are among the causes of oxidative stress in these diseases, whereas hyperglycemia is thought to be the primary cause of oxidative stress in diabetic retinopathy. There is, however, overlap in the molecular mechanisms of ROS production and endogenous protective mechanisms, and therefore treatments targeting oxidative stress could have transferrable benefit.

In this review, a clinical overview of diabetic retinopathy is provided, and then the cellular and mechanistic sources of ROS are discussed, followed by pathological mechanisms that mediate the damage induced by ROS production. Finally, protective mechanisms are discussed, as well as potential therapeutic avenues.

## 2. Diabetic Retinopathy

### 2.1. Epidemiology and Risk Factors

Diabetes mellitus is a chronic, progressive metabolic disease characterized by abnormally raised blood glucose levels which cause multisystem organ damage. Nearly 30 years ago, in 1995, the prevalence of diabetes was estimated to be 4% worldwide and projected to rise to 5.4% in 2025 [14]. Instead, 2021 calculations estimated 537 million people with diabetes, representing 10.5% of the global population. This number is set to further rise to 643 million people in 2030 and 783 million (12.2%) by 2045 [15]. Diabetic retinopathy is a microvascular complication of type 1 and type 2 diabetes and is a leading cause of vision loss worldwide [16,17]. In 2022 among individuals with diabetes, the prevalence of diabetic retinopathy was estimated to be 22% (103 million people), with 6% (28 million) having vision-threatening diabetic retinopathy [18]. The age-standardized prevalence of both moderate–severe vision impairment (visual acuity from <6/18 to 3/60) and blindness (visual acuity of <3/60, or less than 10 degrees of central visual field) due to diabetic retinopathy decreased from 2010 to 2019 [17]. However, the absolute numbers of those with diabetic retinopathy are projected to increase in the next 25 years to 160 million people, with those with vision-threatening diabetic retinopathy rising to 45 million, and those with clinically significant macular edema rising to 28 million [18]. The regional prevalence of diabetic retinopathy among those with diabetes varies worldwide, ranging from 13% in South and Central America (4.5 million people) to 36% in Africa (7 million). The region with the highest absolute numbers of those with diabetic retinopathy is the Western Pacific (31.5 million, prevalence 19%), which also has the highest prevalence of diabetes [18].

The relationship between hyperglycemia and microvascular complications such as diabetic retinopathy in patients with diabetes has been long established [1,2,19,20,21]. Further risk factors for the development and progression of diabetic retinopathy were recently addressed in a systematic review [22]. Increased hemoglobin A1c level, renal impairment, younger age at diagnosis, and increased triglyceride levels were found to be significant factors associated with progression of diabetic retinopathy, as well as increased retinal venular diameter in those with type 1 diabetes. Other factors have less consistent evidence but have been associated with worsening of retinopathy [22]: duration of diabetes [23,24], body mass index, blood pressure [25,26,27], rapid reduction in HbA1c [28,29], sex, and ethnicity.

### 2.2. Classification

Diabetic retinopathy is a progressive disease that, if left untreated, can cause vision loss [30,31]. Due to the stepwise progression of diabetic retinopathy and the optical nature of the eye, diabetic retinopathy is well suited to photographic screening for staging [32], and stratifying screening intervals based on retinopathy grade can be cost effective [33]. Classification systems have evolved to quantify severity of disease and stratify the risk of further progression to enable early detection and treatment. The “gold standard” [34] Early Treatment of Diabetic Retinopathy Study (ETDRS) severity scale graded retinopathy from fundus photographs based on the modified Airlie House classification of diabetic retinopathy [35]. These systems were developed from studies of the natural history of diabetic retinopathy, and they prognosticate the risk of worsening retinopathy and vision loss [36]. The grading system most commonly used worldwide by clinicians [34] is the International Clinical Diabetic Retinopathy Severity Scale [37], a simplified version of the ETDRS scale adapted for clinical use.

### 2.3. Clinical Manifestations and Management

**Figure 1:**
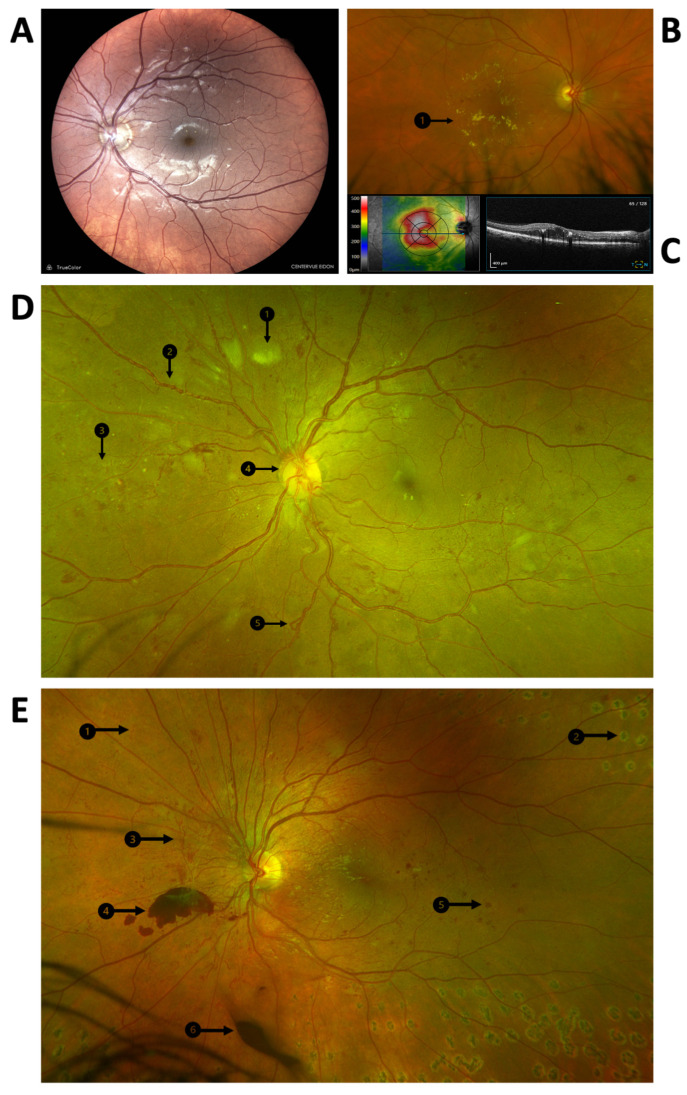
Clinical features of diabetic retinopathy. (**A**) Color photograph of the posterior pole of a left eye demonstrating optic nerve head, vascular arcades, and macula. Diabetic maculopathy without edema is present, and microaneurysms and dot hemorrhages can be seen at the macula. (**B**) Color photograph of the posterior pole of a right eye demonstrating clinically significant diabetic macular edema. Hard exudates appear as well-demarcated yellow deposits at the macula (B1). (**C**) Optical coherence tomography of the right macula that is shown in panel B, demonstrating overall thickening in heat map image on the left and intraretinal fluid on the retinal cross-sectional image on the right. (**D**,**E**) Widefield color fundus photographs of proliferative diabetic retinopathy in two left eyes, demonstrating representative signs of diabetic retinopathy: (D1) cotton wool spot, (D2) venous beading, (D3) intraretinal microvascular abnormalities, (D4) neovascularization at the disc, (D5) venous loop, (E1) intraretinal microvascular abnormalities, (E2) a retinal scar from laser photocoagulation, (E3) retinal neovascularization, (E4) pre-retinal hemorrhage, (E5) blot hemorrhage, and (E6) vitreous hemorrhage.

Diabetic retinopathy can be broadly divided by retinal location. Maculopathy involves the central retina and central visual field (Figure 1A–C). Vision-threatening microvascular complications at the macula may involve ischemia (loss of perfusion) or edema (vascular leakage). Signs of diabetic maculopathy on clinical examination include retinal hemorrhages, microaneurysms, and hard exudates, which are cholesterol precipitates that represent chronic vascular leakage. Macular edema is a significant cause of vision loss in diabetic retinopathy.

Diabetic macular edema is best diagnosed using optical coherence tomography, a non-invasive imaging modality that creates a “optical biopsy” of the macular in cross section. Clinically significant macular edema was defined before the advent of ocular coherence tomography and is diagnosed via slit lamp biomicroscopy as any one of the following: retinal thickening within 500 µm of the center of the macula; hard exudates at or within the center of the macula, if associated with a thickening of adjacent retina; and retinal thickening of at least one disc area, any part of which lies within one disc diameter of the center of the macula [30,38].

Macular grid laser has been used for decades to treat clinically significant macular edema and is superior to no treatment in decreasing the risk of moderate vision loss [30]. The recent use of subthreshold laser as an alternative to treat macular edema and minimize tissue damage has shown a favorable risk–benefit profile in improving visual function [39]. Since the advent of therapeutics targeted against vascular endothelial growth factor (VEGF), macular laser is used less frequently as a monotherapy but is still a viable option for non-center involving macular edema and where access to anti-VEGF treatment is limited [40,41].

The advent of intravitreal anti-VEGF drugs such as ranubizumab and aflibercept heralded an age of unprecedented benefit for those with center-involving diabetic macular edema, but due to short treatment intervals and the need for close monitoring, this created extreme demand on resources [42,43]. A systematic review of anti-VEGF treatment for diabetic macular edema found a significant treatment effect, yielding an improvement in vision of three or more lines of visual acuity to 30–40% of people at one year [44]. New agents such as the anti-angiopoietin-2 and anti-VEGF bispecific antibody purport to offer longer treatment intervals after induction doses [45]. Intravitreal corticosteroids such as triamcinolone acetate are a second-line treatment due to the relatively modest effect in comparison to VEGF blockade, and the elevated intraocular pressure response that occurs in some patients and may require pressure-lowering treatment [41,46,47]. The combination of anti-VEGF and corticosteroid treatments does not yield an advantage over monotherapy [48].

Diabetic retinopathy that involves the peripheral retina follows a stepwise progression from non-proliferative to proliferative diabetic retinopathy which is characterized by the formation of new blood vessels (Figure 1D,E). The clinical signs of diabetic retinopathy show stages of progression. Broadly, retinopathy can be divided into non-proliferative and proliferative disease. Signs of non-proliferative diabetic retinopathy (NPDR) include dot hemorrhages, blot hemorrhages, cotton wool spots, venous beading, and intraretinal microvascular abnormalities. Proliferative diabetic retinopathy (PDR) is characterized by neovascularization of the optic nerve head, the retina, or the anterior segment. Left untreated, fibrovascular complexes may (a) bleed into the posterior chamber of the eye, causing a vitreous hemorrhage, (b) contract and induce a tractional retinal detachment, and (c) extend into the anterior segment of the eye, causing neovascular glaucoma. The complications of PDR are potentially blinding and represent severe end-stage organ damage.

The treatment of peripheral diabetic retinopathy aims to minimize vision loss and to prevent further disease progression. Laser photocoagulation has been the mainstay of treatment for PDR for at least 40 years [30,49]. Prior to laser, the consequence of untreated PDR was legal blindness in half of patients after 5 years [50,51]. Laser photocoagulation, known as panretinal photocoagulation (PRP), involves placing hundreds of laser burns on the peripheral retina. The treatment is highly effective at causing regression of vessels and has been shown to reduce the rate of severe vision loss [52,53,54]. More recently, anti-VEGF drugs have been found to reduce neovascularization in the anterior segment and on the retina in patients receiving injections for diabetic macular edema [55,56]. Further studies confirmed that anti-VEGF therapy is non-inferior to PRP for the treatment of PDR [57]. Recent studies have shown benefit from intravitreal anti-VEGF alone in the treatment of PDR [58,59,60], although visual recovery is slower [61], and patients who are lost to follow-up after anti-VEGF alone are at risk of worse visual outcomes than if they had received laser treatment [62]. The combination of anti-VEGF and PRP is widely used clinically, but the benefit over PRP alone is modest [60].

Non-clearing vitreous hemorrhage or tractional retinal detachment require vitrectomy surgery, which is effective for gaining visual function and stabilizing disease progression [63,64,65,66]. Those patients presenting with worse vision are more likely to require repeat vitrectomy and have a worse visual prognosis [67]. Tractional retinal detachment is an end-stage manifestation of PDR whereby fibrovascular contractile plaques detach the neurosensory retina from the underlying retinal pigment epithelium and requires complex vitrectomy surgery [68]. Preoperative anti-VEGF therapy in the week prior to surgery is helpful to reduce perioperative bleeding and postoperative complications [69].

Proliferative diabetic retinopathy is one of the leading causes of neovascular glaucoma, a secondary glaucoma that represents end-stage disease and is characterized by severe eye pain and extremely high intraocular pressure [70]. This complication is very difficult to manage and, depending on the visual prognosis, may require glaucoma drainage surgery, cyclodestructive procedures, or in the case of a blind painful eye, evisceration or enucleation.

Optical coherence tomography angiography is a new form of retinal imaging that promises to revolutionize the medical management of diabetic retinopathy in the coming years [71,72]. In generating three-dimensional graphics that show the retinal vascular pathology in considerable detail including before it is clinically apparent, the technology provides the opportunity for tailored early intervention. Preclinical diabetic pathology is observed in an area in the central macula or fovea that is devoid of capillaries, known as the foveal avascular zone. Changes include disruption of the foveal avascular zone and capillary dropout, which alter the circular shape of the zone and may be quantified as a circularity index [73].

## 3. Cellular Players in ROS Production

The vascular endothelium has long been a subject of investigation in diabetes given the involvement of the vasculature in diabetic complications. A large body of early work utilized bovine aortic endothelial cells [5,7,74]. These studies showed that culture in high glucose increases the production of ROS, in particular superoxide. Many mechanisms via which hyperglycemia-induced oxidative stress can damage cells were elucidated in these cells.

With regard to studies specifically on retinal endothelial cells, Du et al. showed that primary bovine retinal endothelial cells exhibit elevated superoxide production when cultured in high glucose [10]. Increased cell death induced by high glucose is prevented via the addition of cytoplasmic copper/zinc-dependent superoxide dismutase (SOD1) to the culture media. Many other studies have likewise shown elevated ROS production in bovine retinal endothelial cells or human retinal endothelial cells exposed to high glucose [75,76,77,78,79,80]. Retinal endothelial cells use insulin-independent GLUT1 glucose transporters to both import and export glucose [81]. The susceptibility of retinal endothelial cells to hyperglycemia is proposed to relate to their inability to downregulate GLUT1, and consequently glucose uptake, in the presence of high glucose [82,83]. However, Busik et al. reported a robust series of experiments showing no increase in glucose consumption or ROS production in human retinal endothelial cells cultured in high glucose [84]. These authors showed that high glucose induces expression of interleukin (IL)-1β in other retinal cell types, namely human retinal pigment epithelial cells and Müller cells, and that IL-1β induces superoxide production in human retinal endothelial cells. The authors hypothesized that proinflammatory cytokines such as IL-1β expressed by retinal pigment epithelial and Müller cells in response to hyperglycemia induce oxidative stress in retinal endothelial cells. The current weight of evidence favors the notion that retinal endothelial cells are directly susceptible to glucose-induced oxidative stress in vitro, but other mechanisms including cytokine-induced oxidative stress are also clearly involved. The key pathways inducing oxidative stress at the level of specific cell types in vivo are not yet clear.

Retinal endothelial cells are not the only retinal cell type subject to oxidative stress in diabetes. The immortalized rat retinal Müller cell line, rMC-1, exhibits increased superoxide production in high glucose [10]. Müller cells in the diabetic rat retina exhibit upregulation of heme oxygenase (HO)-1, a marker of oxidative stress, as well as the upregulation of glial fibrillary acidic protein (GFAP), indicating Müller cell activation [85]. Pericytes are another retinal cell type that increases ROS production when cultured under conditions of high glucose concentration [77]. As discussed later, oxidative stress is also induced in pericytes by advanced glycation end products (AGEs), which accumulate in the diabetic retina [86]. This is associated with increased pericyte apoptosis.

In vivo evidence implicates photoreceptors as key players in retinal oxidative stress. Photoreceptors are the greatest producers of ROS in the mouse retina and are predominantly responsible for the increase in retinal superoxide in diabetes [8]. The elevated ROS appear largely restricted to the photoreceptor layer. Early signs of neurodegeneration in diabetic retinopathy are typically observed in the inner retinal layers, although there is some evidence of early photoreceptor apoptosis in diabetic rats [87,88,89]. Nevertheless, photoreceptors seem able to largely withstand their elevated ROS production. Interestingly, retinas with photoreceptor degeneration are protected from the development of diabetic retinopathy, implying an important role in pathology [8,90]. It is suggested that increased photoreceptor metabolism in diabetes, or minor disruptions to oxygen supply, restricts oxygen delivery to the inner retina, resulting in hypoxic ROS production [91]. Indeed, laser photocoagulation, the mainstay treatment for PDR, is proposed to work by destroying photoreceptors in the peripheral retina which decreases oxygen consumption, hypoxic VEGF expression, and neovascularization [92]. However, whether the retina is hypoxic in early diabetes, before degeneration and occlusion of blood vessels, is contentious [93]. Additional research is necessary to conclusively demonstrate how photoreceptors are involved in retinal oxidative stress and the pathogenesis of diabetic retinopathy.

These studies together show that a broad range of retinal cell types likely produce ROS in diabetes. Retinal endothelial cells and pericytes exhibit increased apoptosis in oxidative stress, consistent with pericyte loss and vascular degeneration observed in the course of diabetic retinopathy. The activation of Müller cells and release of proinflammatory factors is likely to disrupt the blood–retinal barrier, clinically evident as fluorescein leakage, and to further contribute to vascular damage. In vivo, the role of direct hyperglycemia versus cytokines, hypoxia, and other stressors as triggers of oxidative stress in different cell types remains unclear. Deeper understanding of the molecular mechanisms of ROS production may open doors to answering these questions, and significant progress has been made in this area.

## 4. Molecular Mechanisms of ROS Production

Superoxide has two major sources in cells of the diabetic retina, mitochondria, and nicotinamide adenine dinucleotide phosphate (NADPH) oxidase (Nox), and both sources contribute strongly to ROS production [8]. In mitochondria, ROS are produced as a natural consequence of electron leakage from the electron transport chain, though production is elevated above the basal level under certain conditions: when the mitochondria are damaged, the mitochondrial membrane potential is high, or the NADH/NAD^+^ ratio in the mitochondrial matrix rises [94]. These conditions are induced by hyperglycemia. Similarly, diabetes and high glucose have been shown to elevate the activity of certain Nox isotypes.

Brownlee eloquently described the process leading from excess glucose to mitochondrial superoxide production as a mechanism unifying disparate pathways of cellular damage in the context of diabetic complications [5,95]. As described by Brownlee, hyperglycemia leads to an oversupply of NADH produced by glucose catabolism as NADH cannot be oxidized rapidly enough by the electron transport chain [96]. Above a threshold NADH/NAD^+^ ratio, there is a sharp increase in the production of superoxide as electrons leak from the electron transport chain to molecular oxygen [97]. Downstream pathways of cell damage are discussed in the following section.

Multiple studies have reported evidence of mitochondrial ROS production in the context of diabetic retinopathy. For example, uncoupling the mitochondrial proton gradient prevents elevated ROS production induced by high glucose in bovine retinal endothelial cells and bovine pericytes [77]. Overexpression of mitochondrial manganese-dependent superoxide dismutase (SOD2) in bovine retinal endothelial cells prevents oxidative stress induced by high glucose [98]. Transgenic overexpression of SOD2 in the mouse retina prevents diabetes-induced oxidative stress [9,99]. Oxidative stress leads to damage to mitochondria, which promotes further ROS production [100,101].

Along with mitochondria, Nox enzymes play a crucial role in ROS production in diabetic retinopathy [75,76]. These membrane-bound enzyme complexes oxidize NADPH to reduce molecular oxygen, forming superoxide [102]. Nox4 expression is elevated in a type 2 diabetic mouse model, as well as in bovine retinal endothelial cells exposed to high glucose or hypoxia [76]. The inhibition of Nox4 ameliorates elevated ROS production and expression of VEGF and hypoxia-inducible factor (HIF)-1α in response to high glucose or hypoxia in bovine retinal endothelial cells. The knockdown of Nox4 in diabetic mouse retinas decreases ROS production and VEGF expression, and it diminishes leakage from retinal vessels [76]. This study shows that ROS production by Nox4 may underlie HIF-1 stabilization and upregulation of VEGF, a key mediator of diabetic retinopathy pathology.

Several interrelated mechanisms of ROS production in diabetic retinopathy have been reported involving the small GTPase Rac1, which is a component of active Nox2. Rac1 activity is promoted in human retinal endothelial cells or diabetic rodent retinas through post-translational modifications such as palmitoylation and prenylation, as well as via the activity of guanine nucleotide exchange factors (GEFs) including Vav2, Tiam1, and Sos1 [75,79,80]. The inhibition of the interaction of Tiam1 or Vav2 with Rac1 prevents high glucose-induced ROS production in retinal endothelial cells [75,79]. A study using bone marrow chimeric mice shows that Nox2 expression by both retinal cells and bone marrow-derived cells is required for elevated adhesion molecule expression, leukocyte binding in the retinal microvasculature, and vessel leakage in diabetes [103].

The adaptor protein p66Shc can activate ROS production by mitochondria and Nox. Human retinal endothelial cells cultured in high glucose show elevated expression of p66Shc [80]. p66Shc disrupts the association of the GEF Sos1 with its partner Grb2 and increases association of Sos1 with Rac1 leading to elevated Rac1 activity and ROS production via Nox2 [80,104]. p66Shc also activates mitochondrial ROS production. p66Shc is phosphorylated by protein kinase C (PKC)-β, which promotes its association with Pin1, enabling its translocation into mitochondria [105]. Here, p66Shc oxidizes cytochrome c to produce hydrogen peroxide [106]. This role of p66Shc in mitochondrial ROS production has been demonstrated in human retinal endothelial cells, and likely also operates in the diabetic rat retina [80]. The p66Shc-mediated production of hydrogen peroxide in mitochondria is associated with the induction of apoptosis.

Many of the studies discussed here report remarkably strong amelioration of ROS production with interventions targeting specific ROS producers. This may reflect a high degree of crosstalk or interdependency between the mechanisms that underpin ROS production. Possibly, multiple ROS producers combine in a synergistic fashion which overcomes defense mechanisms. From a therapeutic perspective, targeted small decreases in ROS production may therefore have a large beneficial impact. However, effective treatment likely also requires reversal of damage induced by ROS, as discussed below.

## 5. Pathological Effects of ROS

### 5.1. Metabolic Dysregulation, AGE Production, and Cell Signaling

ROS damage cells and tissues in multiple ways. They cause direct oxidative damage to macromolecules integral to cell function and survival, and they cause the dysregulation of metabolic pathways, leading to a wide range of effects. There are a set of pathological cellular processes common to various diabetic complications that stem from a unifying pathway of increased oxidative stress [5,95]. These include the activation of PKC, formation of AGEs, upregulation of the receptor for AGEs (RAGE) and its ligands, and elevated flux through the polyol pathway [5,7,74,95]. These are all established mediators of the pathogenesis of complications in diabetes [107,108,109,110]. Mechanistically, the elevated production of superoxide leads to DNA damage and activation of poly ADP-ribose polymerase (PARP) [111]. PARP modifies the glycolytic enzyme glyceraldehyde 3-phosphate dehydrogenase (GAPDH), decreasing its activity. GAPDH can also be inactivated directly under oxidative stress via oxidation of a specific cysteine residue by hydrogen peroxide [112]. Blocking glycolysis at GAPDH causes a build-up of upstream glycolytic intermediates that feed into or activate the noted destructive processes.

The term AGE encompasses various irreversible glycation products formed by non-enzymatic reactions of reducing sugars and their degradation products with proteins, lipids, or nucleic acids [113]. A range of reaction pathways can produce AGEs. Glucose itself is relatively lowly reactive in non-enzymatic glycosylation, but products of glucose metabolism, including dicarbonyls such as methylglyoxal, are highly reactive and play a major role in AGE formation [113,114]. By inhibiting GAPDH, oxidative stress changes the abundance of reaction products of glucose, and it increases the formation of highly reactive dicarbonyls, leading to elevated AGE formation [111]. AGEs drive pathology via multiple mechanisms. They can alter extracellular matrix properties. For example, the inhibition of AGE formation prevents basement membrane thickening on retinal capillaries of diabetic rats [115]. A major means by which AGEs affect cells is by activating receptors such as RAGE. Signaling through RAGE can activate several pathways, including Janus kinase–signal transducer and activator of transcription (JAK-STAT), mitogen-activated protein kinases (MAPKs), phosphatidylinositol-3-kinase (PI3K)/Akt, and Ras/Rac/Cdc42 [116]. AGE production is elevated by ROS, and it can further induce ROS production in a positive feedback loop by activating Nox enzymes [117,118].

It has long been recognized that inhibition of AGE formation limits the progression of experimental diabetic retinopathy in different animals [107,119]. The effects of AGEs are not likely to be limited to one cell type, but certain cell types may be particularly susceptible. Diabetes induces RAGE expression in Müller cells in the rat retina, and high glucose induces RAGE expression in the MIO-M1 human Müller cell line [120]. This upregulation is likely dependent on superoxide production [121]. The inhibition of AGE formation decreases Müller cell activation [85]. In MIO-M1 cells, activation of RAGE induces the expression of proinflammatory and vasoactive factors including IL-6, IL-8, CCL2, and VEGF [120]. Pericytes exhibit decreased proliferation and increased apoptosis when cultured on AGE-modified basement membrane substrate [122]. Incubating cells with SOD (isoform unspecified) or inhibiting Nox blocks oxidative stress and limits the pericyte apoptosis that otherwise follows exposure to AGEs [86,123].

In addition to AGEs, the advanced lipoxidation end-product (ALE) N^ε^-(3-formyl-3,4-dehydropiperidino)lysine (FDP-lysine) accumulates in Müller cells in the diabetic rat retina [124]. FDP-lysine induces apoptosis in MIO-M1 cells, as well as expression of VEGF, IL-6, and tumor necrosis factor (TNF)-α.

The polyol pathway is the two-step conversion of glucose to fructose via sorbitol [125]. Aldose reductase is the enzyme responsible for reduction of glucose to sorbitol, which consumes NADPH. Excess NADPH consumption by aldose reductase has been proposed to exacerbate oxidative stress as NADPH is required to maintain the pool of reduced glutathione [126]. In addition, fructose is a more reactive precursor for AGE formation than glucose [113]. Aldose reductase inhibition is effective in decreasing apoptosis of cultured retinal pericytes and it prevents diabetic retinopathy in rats, suggesting the pathway is an important driver of pathology; however, this therapeutic strategy has seen little success in human patients so far [125,127,128,129].

Another degradation product of glucose that accumulates in hyperglycemia is diacylglycerol (DAG), which is a potent activator PKC-β [130]. PKC-β has various cellular effects [131]. The inhibition of PKC-β prevents vascular dysfunction associated with diabetes in rats [109]. Retinal blood flow is impaired in early diabetic retinopathy partly due to the PCK-mediated suppression of constitutive nitric oxide synthase (eNOS) and decreased nitric oxide production in endothelial cells [132]. The inhibition of PKC-β restores retinal blood flow and decreases leukostasis [109,133]. PKC-β can also facilitate blood–retinal barrier dysfunction via phosphorylation of the tight junction protein occludin, an important mechanism of VEGF-mediated barrier disruption [134,135]. Hyperglycemia induces the activation of nuclear factor kappa-light-chain-enhancer of activated B cells (NF-κB), and this depends on PKC-β [136,137]. NF-κB is well known to be activated as a consequence of oxidative stress, and is a key mediator of inflammatory responses [138]. NF-κB drives expression of proinflammatory cytokines including IL-1β and TNF-α [139]. IL-1β induces superoxide production, activation of MAPKs and proinflammatory caspases, as well as further activation of NF-κB [84]. NF-κB can induce expression of Nox component genes [140]. These observations implicate PKC-β and NF-κB as mediators of proinflammatory signaling in the diabetic retina downstream of oxidative stress.

There are also mechanisms of cell damage beyond these classical pathways. For example, lipid peroxidation leading to ferroptosis is likely a cause of cell death in diabetic retinopathy. Pharmacological inhibition of fatty acid binding protein 4 (FABP4) decreases lipid peroxidation, ROS levels, and iron accumulation in diabetic mouse retinas, and it prevents histological changes [141]. PPAR-γ is partly responsible for the beneficial effects of FABP4 inhibition observed in the ARPE-19 human retinal pigment epithelial cell line exposed to high glucose.

Nitric oxide can react with superoxide to form peroxynitrite, which can damage macromolecules. Knockout of inducible nitric oxide synthase (iNOS) protects the mouse retina against elevated superoxide production, capillary degeneration, the formation of pericyte ghosts, and leukostasis [142]. The mechanism by which iNOS promotes superoxide production was not assessed in the referenced study, but may relate to nitric oxide-mediated damage to electron transport chain components.

### 5.2. Lasting Damage and Metabolic Memory

Good glycemic control greatly reduces the occurrence of diabetic retinopathy [1]. However, a temporary period of poor glycemic control bestows a higher chance of retinopathy progression long after institution of intensive glycemic control [143,144,145]. This effect is termed metabolic memory. It indicates that retinal cells are damaged by transient metabolic dysregulation in a way that persists after the normalization of systemic metabolism. In patients, the metabolic memory effect is still evident over 10 years after institution of intensive glycemic control [145]. The mechanisms of metabolic memory remain subject to research but significant advances have been made, with evidence of mitochondrial damage, and epigenetic and signaling changes. Oxidative stress persists following transient exposure to high glucose, and plays a key role in metabolic memory [146].

Firstly, transient hyperglycemia damages mitochondria. Markers of oxidative and nitrative stress are elevated; mitochondrial superoxide is elevated; glutathione is decreased; and mitochondrial DNA is damaged and carries more oxidative modifications in the retinas of rats subject to poor followed by good glycemic control, compared to rats with continuous good control [100,101]. Oxidative DNA damage repair enzymes are downregulated. Mitochondrial gene expression and the activity of the electron transport chain complex III are lower. Interestingly, matrix metalloproteinase 9 (MMP9) may be a key mediator of mitochondrial damage [147].

Secondly, transient hyperglycemia induces lasting epigenetic changes. In human aortic endothelial cells, hyperglycemia induces H3K4me1 methylation of the NF-κB p65 promoter region via the histone methyltransferase SetD7 [148]. This causes persistent upregulation of NF-κB p65 and, subsequently, persistent upregulation of proinflammatory proteins including CCL2, IL-6, and NOS2. Adhesion molecules including intercellular adhesion molecule 1 and vascular cell adhesion molecule 1 are also induced. Importantly, this process depends on superoxide production. SetD7 is also implicated in H3K4me1 methylation at the *KEAP1* gene promoter in bovine retinal endothelial cells transiently exposed to high glucose [149]. This increases Kelch-like ECH-associated protein (KEAP)1 expression. As detailed later, KEAP1 represses the activity of the antioxidant transcription factor nuclear factor erythroid 2-related factor2 (NRF2). As another example, methylation of a key regulatory region of the *Gclc* gene promoter is altered in the diabetic rat retina in a way that decreases glutamate-cysteine ligase catalytic subunit (GCLC) expression, and this persists after good glycemic control [150]. GCLC is a key enzyme involved in the synthesis of glutathione, so this is likely to impede cellular defense against oxidative stress.

Nuclear DNA is not the only target of change. Mitochondrial DNA, particularly the D-loop region, is hypermethylated in bovine retinal endothelial cells by the DNA methyltransferase Dnmt1 in response to high glucose [151]. High glucose-induced elevated apoptosis is prevented by Dnmt1 blockade. The key findings hold true in donor retinas from diabetic patients.

Finally, there is evidence of persistent signaling changes that decrease the glucose level required to elevate ROS production. The localization of the glycolytic enzyme hexokinase 2 (HK2) to the mitochondrial outer membrane via binding to the voltage-dependent anion channel (VDAC) is required for efficient ATP-ADP transfer dynamics in respiration, and inhibition of this interaction elevates ROS production [152,153,154]. In human aortic endothelial cells, transient hyperglycemia causes a cascade from increased protein phosphatase 2A (PP2A) activity to decreased Akt activity, increased glycogen synthase kinase (GSK)-3β activity and increased VDAC phosphorylation, which disrupts the interaction of VDAC with HK2 [153]. High PP2A activity likely originates with the modification of PPA2 by hydroxyl radicals generated by the reaction of hydrogen peroxide with free iron, which is preceded by elevated superoxide production. The altered HK2-VDAC interaction renders cells persistently susceptible to ROS production at normal glucose levels.

## 6. Regulation of Oxidative Stress by Non-Coding RNA

Non-coding RNAs (ncRNAs) are a diverse group of RNA transcripts ranging in length from 20 nucleotides to more than 1 kilobase, which regulate the expression of many genes by directly affecting DNA and histone methylation, transcription, and translation [155,156,157]. Non-coding RNA includes microRNA (miRNA), long non-coding RNA (lncRNA), circular RNA (circRNA), and short nucleolar RNA host genes (SNHG/snoRNA). After post-transcriptional processing, and formation of the microRNA-induced silencing complex (mRISC), miRNA binding to mRNA leads to translational repression and degradation of the targeted transcript [158]. While lncRNA have many functions, they play an important regulatory role as competing endogenous RNA (ceRNA), where lncRNA competitively bind miRNA, acting as a molecular “sponge” and altering the regulation of the target transcripts [159]. Other ncRNA such as circRNA, covalently closed RNA loops, and SNHG, when processed post-transcriptionally into lncSNHG RNA, can act in a similar manner [160,161]. The emerging role for ncRNA in the induction and progression of oxidative stress in the diabetic retina has been the subject of much recent study [162,163,164].

MicroRNA regulate the expression of many gene transcripts in the context of diabetic retinopathy, and the connection between miRNA and oxidative stress in diabetic retinopathy is well established [165]. New research has implicated miRNA regulation in the mechanism of antioxidant activity of plant extracts [166,167], and flavonol compounds [168] in the ARPE-19 cell line. An investigation of the antioxidant activity of 25-hydroxyvitamin D3 (vitamin D) in human retinal endothelial cells revealed a reduction in miR-93 transcript with high glucose and vitamin D treatment, compared to high glucose alone, as well as reduced ROS production, malondialdehyde (MDA) content, and an increased level of reduced glutathione (GSH) [169]. Another investigation using a rat model of streptozotocin-induced diabetes indicated that miR-93 targeted sirtuin 1 (SIRT1) in the diabetic retina, inducing changes indicative of oxidative stress and inflammation which could be reversed by SIRT1 overexpression [170]. Several other miRNAs have recently been implicated in oxidative damage in diabetic retinopathy, including miR-1, miR-19a, and miR-320a repression of mitochondrial SIRT transcripts SIRT3, SIRT4, and SIRT5, respectively [171], miR-301a-3p regulation of six-transmembrane epithelial antigen of prostate 4 (STEAP4) expression [172], and miR-338-3p negative regulation of glutamine transporter SLC1A5 expression, leading to ferroptosis [173].

Conversely, the expression of several miRNAs may protect against oxidative damage, as indicated by recent publications. One article described decreased levels of miR-132 and miR-126 in the plasma of patients with NPDR compared to control patients with diabetes but no retinal disease [174]. A significant inverse correlation between miR-126 and plasma MDA levels, an indicator of oxidative stress, was also observed. At the cellular level, work using ARPE-19 cells and human umbilical vein endothelial cells (HUVEC) indicated that miR-205-5p expression is downregulated by ROS, and in the presence of ROS, the overexpression of miR-205-5p reduced VEGFA expression by the ARPE-19 cells [175]. Furthermore, HUVEC grown in conditioned medium from miR-205-5p mimic-transfected ARPE-19 cells showed reduced angiogenesis in tube formation assays. Another article indicated that the overexpression of miR-139-5p in ARPE-19 cells reduced Lim-only factor 4 transcript levels and reversed indicators of oxidative stress following high glucose treatment [176].

An emerging area of investigation is the regulation of transcription at distant or adjacent sites by miRNA packaged in exosomes [177], and research indicates this phenomenon may be relevant to oxidative damage in diabetic retinopathy. As described in two recent publications, mouse retinal microvascular endothelial cells or Müller cells were treated with high glucose, followed by exosomes derived from bone marrow mesenchymal stem cell transfected with miR-133-3p or miR-486-3p, respectively [178,179]. The investigators observed that exosome treatment increased SOD and catalase activity, and it reduced glutathione content in high glucose-treated Müller cells [178]; SOD, catalase and glutathione peroxidase activity were increased, and MDA content was decreased in high glucose-treated endothelial cells exposed to exosomes [179]. Another recent study suggested the miR-17-3p from human umbilical mesenchymal stem cell-derived exosomes reduced oxidative damage, apoptosis, and inflammation in the retinas of diabetic mice [180]. A summary of the microRNA discussed in this section is presented in Table 1.

Numerous lncRNA have been linked to oxidative damage in diabetic retinopathy, including metastasis-associated lung adenocarcinoma transcript 1 (MALAT1), Hox antisense intergenic RNA (HOTAIR), nuclear paraspeckle assembly transcript 1 (NEAT1), Arid2-IR, OGRU, and HIF1 antisense RNA 2 (HIF1A-AS2) [181,182,183,184,185,186]. For example, high glucose-driven increase in expression of MALAT1 in human retinal endothelial cells resulted in increased KEAP1 levels and subsequent cytoplasmic sequestration of NRF2 [182]. The reduction of MALAT1 by siRNA reduced KEAP1 expression and led to an increase in NRF2-responsive antioxidant defense genes *HO-1* and *SOD2* transcripts. As further evidence of MALAT1 involvement in oxidative stress in diabetic retinopathy, ARPE-19 cells treated with high glucose also up-regulated MALAT1 transcript, as well as increased the expression of multiple proinflammatory cytokines including TNF-α and CCL2 via IGF2BP2-dependent activation of NF-κB [187]. Treatment with the antioxidant ascorbic acid reduced both MALAT1 expression and proinflammatory cytokine expression in high glucose-treated ARPE-19 cells. High glucose-dependent upregulation of HOTAIR in human retinal endothelial cells, as well as increased HOTAIR levels in serum and vitreous samples from patients with diabetic retinopathy [183]. A reversal of indicators of oxidative stress followed siRNA knockdown of HOTAIR and high glucose treatment of human retinal endothelial cells: an increase in mitochondrial depolarization and reduced levels of 8-OHdG, a marker of oxidative DNA damage.

In contrast, several lncRNA have been identified as playing protective roles during oxidative stress in diabetic retinopathy. One very recent study indicated that the level of lncRNA transmembrane phosphatase with tensin homologue pseudogene 1 (TPTEP1) was reduced in diabetic retinopathy patient serum and in human retinal endothelial cells after high glucose treatment [188]. TPTEP1 was identified as a competing endogenous RNA for miR-489-3p, which in turn targeted NRF2 transcript. Overexpression of TPTEP1 reduced ROS production and increased the expression of antioxidant proteins in human retinal endothelial cells following high glucose treatment. Another study using RNA-sequencing identified regulatory changes in 16 lncRNA following H_2_O_2_ treatment of HUVEC. [189]. Further experiments showed that the expression of one of these, the short nuclear host gene lncRNA SNHG16, was repressed by AGE, high glucose, and H_2_O_2_ treatment in human retinal endothelial cells. SNHG16 was shown to interact with miR-195, and restoring the expression of SNHG16 reduced oxidative stress-induced HREC tube formation. A similar study of the lncRNA FLG antisense RNA1 (FLG-AS1) demonstrated reduced ROS production and MDA levels, and increased SOD1 activity in high glucose-treated ARPE-19 cells overexpressing FLG-AS1 [190]. Reduced retinal injury was also observed in diabetic rats receiving a FLG-AS1 lentiviral expression construct by intraocular injection.

Circular RNAs have also been implicated in the pathophysiology of oxidative damage in diabetic retinopathy [191,192,193,194]. Studies demonstrated ceRNA activity of three separate circRNA, i.e., circFTO (targeting miR-148-3p in ARPE-19), circSLC16A12 (targeting miR-140-3p in HREC), and circRNA_0084043 (targeting miR-140-3p in ARPE-19), and knockdown of these circRNAs reduced MDA content and increased SOD and/or glutathione peroxidase activity, indicating a reduction in oxidative stress [195,196,197]. Interestingly, miR-140-3p is common between retinal pigment epithelial cells and retinal endothelial cells. Although the transcripts regulated by this miRNA differ between the two cell types, both are growth factors: transforming growth factor (TGF)-α and fibroblast growth factor 2. TGF-α expression is also increased in high-glucose-treated retinal pigment epithelial cells by circFTO via sponging of a different miRNA, miR-148-3p. Taken together, these results suggest that circRNA-mediated dysregulation of growth factor expression in diabetic retinopathy warrants further attention. A summary of the lncRNA and circRNA discussed in this section is shown in Table 2. 

## 7. Protective Mechanisms and Therapeutic Avenues

Cells possess protective mechanisms to restrict the production of ROS and to convert highly reactive species into less reactive species. However, these mechanisms are not limitless, and there is evidence they are suppressed or overcome in diabetic retinopathy. Interventions that supplement, restore, or augment endogenous antioxidant defenses provide promising therapeutic avenues. The reversal of lasting damage may also be needed to counter metabolic memory.

Superoxide is eliminated by SOD, which catalyzes its conversion to hydrogen peroxide. Though still a reactive species, hydrogen peroxide is less reactive than superoxide, and it is important in normal cell signaling [6]. Hydrogen peroxide can be further degraded to water by catalase or peroxidases. SOD2 activity is decreased in the diabetic rat retina [98]. The overexpression of SOD2 in transgenic mice protects against the development of acellular retinal capillaries in diabetes [9]. It prevents the diabetes-induced increase in retinal superoxide, blocks increased mitochondrial membrane permeability, and maintains electron transport chain complex III activity [9,99]. This indicates that the restoration of SOD2 activity is likely to be protective.

The uncoupling of the mitochondrial proton gradient from ATP production decreases ROS production by mitochondria. Uncoupling proteins UCP1-3 are activated by superoxide to limit its further production [198]. UCP1 and UCP2 are reportedly elevated at the RNA level by moderately high glucose (23 mM) in cultured bovine retinal endothelial cells and pericytes, but this effect is lost at excessively high glucose levels (30 mM) [77]. It has been hypothesized that uncoupling may be elevated as a protective mechanism but overcome under severe oxidative stress, although this hypothesis requires validation. UCP2 is reportedly downregulated in the diabetic rat retina, and its expression is restored by losartan, a blocker of angiotensin II type 1 receptor [199]. Interestingly, mouse retinal mitochondria, particularly in photoreceptors which exhibit an extraordinary respiratory rate, are unusually highly uncoupled from ATP synthesis even in the non-diabetic retina [200]. A high degree of uncoupling in health may render this protective mechanism particularly susceptible to being overcome in diabetes.

The glutathione system is a major endogenous means of protection from oxidative stress. The reduced form of glutathione (GSH) directly scavenges ROS and acts as a cofactor for enzymatic elimination of ROS [201]. The oxidized dimeric form of glutathione (GSSG) is produced in these reactions. Glutathione reductase uses NADPH to reduce GSSG back to GSH. The level of GSH is decreased in the retinas of diabetic rats, in mitochondria in the retinas of diabetic mice, and in pericytes cultured in high glucose concentrations [9,100,127]. As stated above, the transcription of a key enzyme in glutathione synthesis, GCLC, is inhibited in the diabetic rat retina due to epigenetic changes in the promoter [150]. These observations indicate the glutathione system is likely stressed in its ability to scavenge ROS in the diabetic retina.

The transcription factor NRF2 is integral to the cellular response to oxidative stress [202]. It also has anti-inflammatory effects [203]. Classically, in the non-stressed state, KEAP1, as an adaptor protein for an E3 ubiquitin ligase, directs NRF2 for polyubiquitination and degradation. Under stress, the interaction of KEAP1 with NRF2 is disrupted, allowing NRF2 to translocate to the nucleus, heterodimerize with partners, and bind to antioxidant response elements to transactivate expression of protective target genes. These genes include *GCLC*, as well as *HO-1*, *GSR* (encoding glutathione reductase), and *SOD1* [204,205,206].

In the rat, NRF2 is expressed highly in the retina relative to the brain and liver [207]. It is expressed in several cell types in human and mouse retinas, and especially highly in Müller cells [208]. In one report, NRF2 expression in the rat retina transiently decreased in early diabetes before increasing above the non-diabetic level [207]. In another, the retinal NRF2 level was significantly lower than controls after a longer period of diabetes [209]. While the specifics of NRF2 expression and activity in diabetic retinopathy remain to be clarified, most evidence points towards its decreased activity on the basis of downregulation of targets under its control. Important recent reports have shown that decreased NRF2 activity in the diabetic mouse retina is due to a KEAP1-independent mechanism of degradation which instead involves the stress response protein regulated in development and DNA damage responses 1 (REDD1) [210,211]. REDD1 activates GSK-3β-dependent phosphorylation of NRF2, which targets it for degradation [210,212,213]. It is clear that NRF2 plays an important protective role in diabetic retinopathy. The knockout of NRF2 in a mouse model of diabetic retinopathy increases TNF-α expression, causes earlier blood–retinal barrier breakdown, and exacerbates functional defects [208]. Consistent with this, strong protective effects of NRF2 activation have been reported in animal models, and this is an exciting avenue for future therapies [209,214].

The multifunctional deacetylase SIRT1 is also involved in protection against diabetic retinopathy [215,216]. It can deacetylate transcription factors such as NF-κB p65 and Forkhead box protein O1 (FOXO1), which decreases their activity and has beneficial effects in countering inflammation and oxidative stress. SIRT1 also represses the expression of p66Shc, an effect which is likely to decrease oxidative stress by limiting Rac1 and Nox2 activation [217]. SIRT1 expression is downregulated in the diabetic mouse retina, and its overexpression protects against the development of diabetic retinopathy [215]. SIRT1 is regulated in the diabetic retina by regulatory RNAs, as discussed above. The restoration of SIRT1 activity has promise not only for preventing diabetic retinopathy development but also for reversing damage, given its epigenetic mode of action.

With the wealth of evidence for the role of oxidative stress in diabetic retinopathy, particularly in cell culture and animal models, oxidative stress has been suggested as a strong candidate target for therapeutics. Consequently, many antioxidants have been trialed in animals and in human diabetic patients. The analysis of specific treatments is beyond the scope of this review. For recent comprehensive reviews on antioxidant therapeutics in the treatment of diabetic retinopathy, see references [218,219,220]. Potential therapeutics include antioxidant enzyme cofactors, as well as non-enzymatic antioxidant compounds and formulations, including polyphenols, flavonoids, carotenoids, and certain vitamins, among other molecules. However, although certain compounds have shown significant benefits in cell culture and in animal models, as well as limited benefits in humans in some instances, therapeutics targeting oxidative stress have yet to see successful translation to clinical use [218].

In a recent systematic review of oral antioxidant supplementation in patients with diabetic retinopathy, it was noted that multiple formulations produce benefits in biochemical parameters such as plasma antioxidant capacity and lipid peroxidation, but there are limited benefits to function and clinical course [218]. One reason for the limited efficacy observed thus far may be that the pathological course of diabetic retinopathy may be quite advanced before it is clinically evident. The advent of new technologies such as optical coherence tomography angiography provides the capability to detect the first signs of disease in patients. Early and long-term use of antioxidants has been convincingly shown to be beneficial in animal studies [221]. The window of opportunity for maximum benefit of antioxidants is likely to be quite early, in concert with control of blood sugar, and this warrants assessment in clinical trials. It is probable that at the later stages of disease, antioxidants have less capacity to alter the clinical course due to the accumulation of lasting damage to intracellular macromolecules, organelles, and the vascular structures. At later stages, antioxidants may be most effective if partnered with treatments that aim to reverse lasting epigenetic and signaling damage. The mechanisms of lasting damage and strategies for their reversal should be target areas for research. New findings in these areas could pave the way for therapeutics that are effective at later stages of disease.

## 8. Conclusions

There is abundant evidence for the role of oxidative stress in the pathogenesis of diabetic retinopathy. A range of stressors, including hyperglycemia, hypoxia, and cytokines, are able to induce oxidative stress in retinal cells. The specific stressors acting most strongly on each cell type in vivo remain less clear. It is possible that hyperglycemia directly induces oxidative stress in each cell type. Alternatively, hypermetabolic photoreceptors may induce an oxygen deficit in inner retinal layers, leading to hypoxia-induced oxidative stress. In either case, once oxidative stress arises, it causes metabolic derangements and the accumulation of toxic products such as AGEs. Importantly, it also causes lasting damage in the form of epigenetic modifications, DNA damage in the nucleus and mitochondria, and alterations to signaling pathways controlling ROS production. Sufficiently advanced, such damage likely renders oxidative stress self-sustaining. Cell apoptosis and dysfunction as a result of these mechanisms is proposed to cause the clinical features of diabetic retinopathy, ranging from neurodegeneration to vascular leakage, and vessel degeneration.

The retina is likely prone to oxidative stress relative to other tissues given its high metabolic activity, and this is reflected in the high basal activity of antioxidant pathways such as mitochondrial uncoupling and the NRF2 transcription factor. In diabetes, mechanisms of protection against oxidative stress are downregulated or overcome, permitting oxidative damage. Therapeutics targeting oxidative stress hold great potential in the treatment of diabetic retinopathy but have yet to enter clinical practice. It is possible that scavenging-type antioxidants must be utilized early in the course of disease, prior to the development of lasting damage. Antioxidants that augment the natural mechanisms of ROS protection, and the development of treatments that specifically target mechanisms of lasting damage, or which reverse such damage, are promising avenues for advancements.

## Figures and Tables

**Table 1:** Examples of microRNA (miRNA) implicated in diabetic retinopathy.

miRNA	Regulation in Diabetic Retinopathy	Target(s)	Contribution to Diabetic Retinopathy Pathology	Reference(s)
miR-138-5p	Upregulated	SIRT1	Increased ferroptosis	[166]
miR-29b	Downregulated	PTEN	Increased oxidative stress and apoptosis	[167]
miR-34a	Upregulated	P53	Increased oxidative stress and apoptosis	[168]
miR-93-5p	Upregulated	SIRT1	Increased oxidative stress and inflammation	[169,170]
miR-1	Upregulated	SIRT3	Increased oxidative stress	[171]
miR-19b	Upregulated	SIRT4	Increased oxidative stress	[171]
miR-320	Upregulated	SIRT5	Increased oxidative stress	[171]
miR-301a-3p	Upregulated	STEAP4	Increased oxidative stress and apoptosis	[172]
miR-338-3p	Upregulated	SLC1A5	Increased oxidative stress and ferroptosis	[173]
miR-126	Downregulated	SIRT1, SPRED, CRK, PLAGL	Potential biomarker	[174]
miR-132	Downregulated	SIRT1, SPRED1, CRK, PLAGL2	Potential biomarker	[174]
miR-205-5p	Downregulated	VEGFA	Increased angiogenesis	[175]
miR-139-5p	Downregulated	LMO4	Increased oxidative stress and inflammation	[176]
miR-133-3p	Downregulated	FBN1	Increased oxidative stress and angiogenesis	[178]
miR-486-3p	Downregulated	TLR4	Increased oxidative stress and inflammation	[179]
miR-17-3p	Downregulated	STAT1	Increased oxidative stress and inflammation	[180]

Abbreviations: SIRT1: Sirtuin 1; PTEN: phosphatase and tensin homolog; P53: tumor protein P53; SIRT3: Sirtuin 3; SIRT4: Sirtuin 4; SIRT5: Sirtuin 5; STEAP4: six-transmembrane epithelial antigen of prostate 4; SLC1A5: solute carrier family 1 member 5; SPRED: sprout related EVH domain containing 1; CRK: CRK proto-oncogene, adaptor protein; PLAGL2: PLAG1 like zinc finger 2; VEGFA: vascular endothelial growth factor A; LMO4: LIM-only factor 4; FBN1: fibrillin 1; TLR4: Toll-like receptor 4; STAT1: signal transducer and activator of transcription 1.

**Table 2:** Examples of long non-coding RNA (lncRNA) and circular RNA (circRNA) implicated in diabetic retinopathy.

ncRNA	Type	Regulation in Diabetic Retinopathy	Target(s)	Contribution to Diabetic Retinopathy Pathology	Reference(s)
MALAT1	lncRNA	Upregulated	KEAP1 IGF2BP3	Increased oxidative stress and apoptosis	[182,187]
HOTAIR	lncRNA	Upregulated	Epigenetic regulation	Increased oxidative DNA damage and angiogenesis	[183]
NEAT1	lncRNA	Upregulated	TGFβ1, VEGFA	Increased oxidative stress and inflammation	[184]
HIF1-AS2	lncRNA	Upregulated	MAPK, VEGFA	Increased oxidative stress and DNA damage	[185]
ARID2-IR	lncRNA	Upregulated	Smad3	Increased oxidative stress and inflammation	[181]
OGRU	lncRNA	Upregulated	miR-320-3p, USP14	Increased oxidative stress and inflammation	[186]
TPTEP1	lncRNA	Downregulated	miR-489-3p, NRF2	Increased oxidative stress	[188]
SNHG16	lncRNA	Downregulated	miR-195, MFN2	Increased angiogenesis	[189]
FLG-AS1	lncRNA	Downregulated	miR-380-3p, SOCS6	Increased oxidative stress and inflammation	[190]
circUBAP2	circRNA	Upregulated	miR-589-5p, EGR1	Increased oxidative stress and angiogenesis	[191]
circADAM9	circRNA	Upregulated	miR-338-3p, CARM1	Increased oxidative stress and apoptosis	[192]
circ_0041795	circRNA	Upregulated	miR-589-5p, YAP1	Increased oxidative stress and inflammation and apoptosis	[193]
circ_0000615	circRNA	Upregulated	miR-646, YAP1	Increased ROS production and apoptosis	[194]
circFTO	circRNA	Upregulated	miR-148-3p, TGFA	Increased oxidative stress and inflammation	[195]
circSLC16A12	circRNA	Upregulated	miR-140-3p, FGF2	Increased oxidative stress and angiogenesis and apoptosis	[196]
circ_0084043	circRNA	Upregulated	miR-140-3p, TGFA	Increased oxidative stress and inflammation	[197]

Abbreviations: MALAT1: metastasis-associated lung adenocarcinoma transcript 1; KEAP1: Kelch-like ECH-associated protein 1; IGF2BP3: insulin-like growth factor 2 mRNA binding protein 3: HOTAIR: Hox antisense intergenic RNA; NEAT1: nuclear paraspeckle assembly transcript 1; TGFβ1: transforming growth factor beta 1; VEGFA: vascular endothelial growth factor A; HIF1-AS2: HIF1A antisense RNA 2; MAPK: mitogen-activated protein kinase 1; ARID2-IR: AT-rich interaction domain 2-intergenic region; Smad3: SMAD family member 3; USP14: ubiquitin-specific peptidase 14; TPTEP1: transmembrane phosphatase with tensin homologue pseudogene 1; NRF2: nuclear factor erythroid 2-related factor; SNHG16: small nucleolar RNA host gene 16; MFN2: mitofusin 2; FLG-AS1: FLG antisense RNA 1; SOCS6: suppressor of cytokine signaling 6; EGR1: early growth response 1; CARM1: coactivator-associated arginine methyltransferase 1; YAP1: yes-associated protein 1; TGFA: transforming growth factor alpha; FGF2: fibroblast growth factor 2.

## Data Availability

Not applicable.
